# Sex disparities in mortality and cardiovascular outcomes in chronic kidney disease

**DOI:** 10.1093/ckj/sfae044

**Published:** 2024-02-21

**Authors:** Olga Balafa, Beatriz Fernandez-Fernandez, Alberto Ortiz, Evangelia Dounousi, Robert Ekart, Charles J Ferro, Patrick B Mark, Jose M Valdivielso, Lucia Del Vecchio, Francesca Mallamaci

**Affiliations:** Department of Nephrology, University Hospital of Ioannina, Ioannina, Greece; Department of Nephrology and Hypertension, IIS-Fundacion Jimenez Diaz UAM, Madrid, Spain; Department of Nephrology and Hypertension, IIS-Fundacion Jimenez Diaz UAM, Madrid, Spain; Nephrology Dept, Faculty of Medicine, University of Ioannina and University Hospital of Ioannina. Ioannina, Greece; Department of Dialysis, Clinic for Internal Medicine, Faculty of Medicine, University Medical Centre Maribor, Maribor, Slovenia; Institute of Cardiovascular Sciences, University of Birmingham, Birmingham, UK; School of Cardiovascular and Metabolic Health, University of Glasgow, Glasgow, UK; Vascular and Renal Traslational Research Group, UDETMA, Biomedical Research Institute of Lleida, IRBLleida, Lleida, Spain; Department of Nephrology and Dialysis, Sant'Anna Hospital, ASST Lariana, Como, Italy; Department of Nephrology, Dialysis, and Transplantation Azienda Ospedaliera ‘Bianchi-Melacrino-Morelli’ & CNR-IFC, Reggio Calabria, Italy

**Keywords:** cardiovascular disease, chronic kidney disease, gender, mortality, sex

## Abstract

Sex (biologically determined) and gender (socially constructed) modulate manifestations and prognosis of a vast number of diseases, including cardiovascular disease (CVD) and chronic kidney disease (CKD). CVD remains the leading cause of death in CKD patients. Population-based studies indicate that women present a higher prevalence of CKD and experience less CVD than men in all CKD stages, although this is not as clear in patients on dialysis or transplantation. When compared to the general population of the same sex, CKD has a more negative impact on women on kidney replacement therapy. European women on dialysis or recipients of kidney transplants have life expectancy up to 44.8 and 19.8 years lower, respectively, than their counterparts of similar age in the general population. For men, these figures stand at 37.1 and 16.5 years, representing a 21% to 20% difference, respectively. Hormonal, genetic, societal, and cultural influences may contribute to these sex-based disparities. To gain a more comprehensive understanding of these

differences and their implications for patient care, well-designed clinical trials that involve a larger representation of women and focus on sex-related variables are urgently needed. This narrative review emphasizes the importance of acknowledging the epidemiology and prognosis of sex disparities in CVD among CKD patients. Such insights can guide research into the underlying pathophysiological mechanisms, leading to optimized treatment strategies and ultimately, improved clinical outcomes.

## INTRODUCTION

There is a growing interest in studying sex disparities in various research fields, including medicine and health [1]. Before delving into further detail, the difference between sex and gender must be highlighted. The terms sex and gender are often used interchangeably in both the lay and medical literature. However, whereas sex refers to a set of biological attributes associated with physical and physiological features, gender refers to the social constructed roles, behaviours, expressions, relationships, and identities of men and women [2].

**Figure 1:**
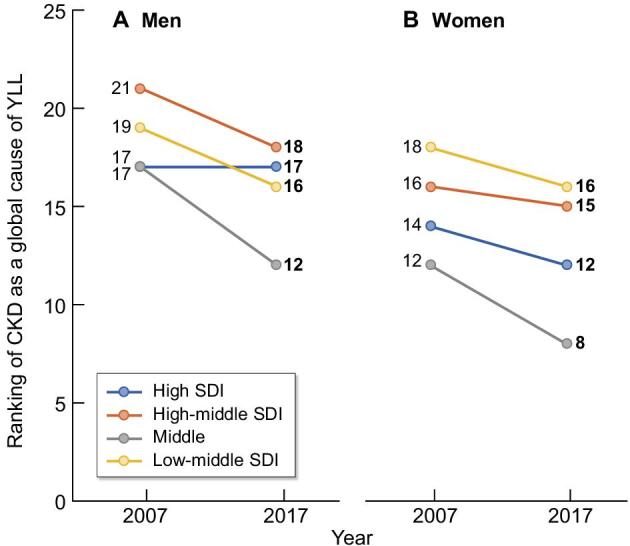
Ranking of chronic kidney disease as a global cause of years of life lost (YLL) among GBD cause hierarchy level 3 causes in men (**A**) and in women (**B**) in 2007 and 2017, according to Socio-Demographic Index (SDI) and [5]. The figure representing a ranking, a lower numerical value reflects a larger negative impact of CKD on YLL. From 2007 to 2017, the mean ranking position changed from 18.5 to 15.75 in men and from 15 to 12.75 in women, i.e. the contribution of CKD to global YLLs is increasing.

While medical research has historically focused mainly on male subjects, there is now recognition that women may respond differently to certain treatments or interventions. Cardiologists have a wide number of studies focused on sex disparities in cardiovascular diseases (CVD) [3], whereas other specialties, such as nephrology, have explored sex differences to a lesser extent. Nevertheless, evidence suggests that the progression of chronic kidney disease (CKD) is influenced by sex [4], and a crucial concern is the under-representation of women in clinical trials, despite constituting more than half of the world's population and CKD being a more common global cause of years of life-lost (YLL) in women than in men [5] (Fig. 1).

CKD is a global health challenge [6] ranking as the ninth top cause of female deaths in the USA but not among the top ten leading causes of death in men [7]. Despite the higher prevalence of CKD in women [8], its progression is faster in men [9]. Men also undergo kidney replacement therapy (KRT)—dialysis or renal transplantation—more often than women [10, 11]. Although the identification, monitoring, and management of most people with CKD happens in primary care, evidence of differences by sex primarily stems from the minority of patients referred to nephrology specialist units. Notably, despite women being more frequent kidney donors, they have on a global scale a lower probability of receiving kidney transplants [12]. Consequently, investigating sex disparities in CKD patients is both an academic endeavour and an ethical and societal imperative. This review emphasizes the need to prioritize research into sex disparities in the epidemiology and prognosis of CVD among CKD patients, aiming to improve therapy and outcomes.

## SEX DISPARITIES IN THE INCIDENCE AND PROGRESSION OF CKD

**Figure 2:**
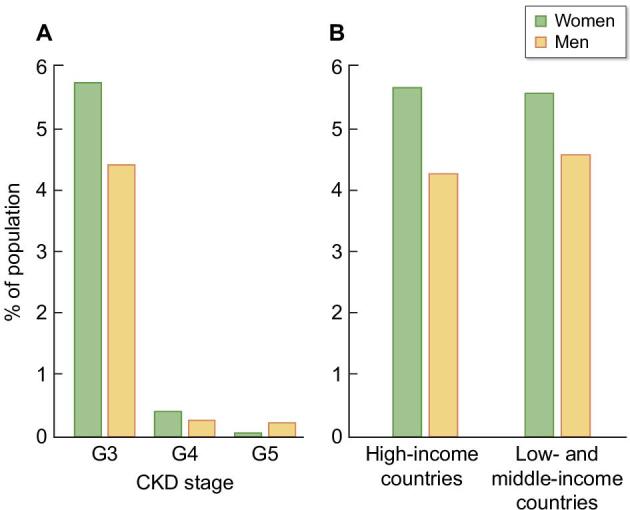
Prevalence of adult population (female and male) with CKD. (**A**) Prevalence % of USA population with CKD stages G3, 4 and 5 in time period 2017 to March 2020. (**B**) Age-standardized prevalence % of population with CKD stages 3–5 in high and low/middle-income countries (data from [13] and [15]).

Over the years, epidemiological data have consistently reported a higher prevalence of CKD in females than in males [4, 13]. In this respect, the Global Burden of Disease (GBD) study showed a higher percentage of females having CKD worldwide, independently of the socio-demographic index (SDI) of the geographic area [14] (Fig. 2). This was also true when considering the age-standardized prevalence of CKD (1.29 times higher in females) [15]. Another example comes from the National Health and Nutrition Examination Survey (NHANES) in the United States. Data from 7137 subjects in the 1999–2014 population showed that females represented 56–59% of individuals having CKD across all CKD stages [16]. The percentage was even higher when considering only subjects over 65 years old [17].

Conversely, epidemiological data show higher prevalence of the male sex among those receiving KRT. According to the GBD, the global age-standardized incidence of dialysis and transplantation is 1.47 times greater among males than among females [14]. Similar observations are available from registry data [13, 18].

Several reasons have been proposed to explain this discrepancy. First, considering that formulae for glomerular filtration rate (GFR) estimation contain serum creatinine, they could perform differently in the two sexes and over-diagnose CKD in females. This methodological aspect has been partially improved by using the CKD-EPI formula [19]. Moreover, females have a longer life expectancy and thus more time to develop CKD compared to males. Conversely, especially in low-income countries, women may have reduced access to expensive treatments such as dialysis and thus receive conservative care more often [20].

Apart from these epidemiological considerations, various observations indicate that GFR declines faster in males than in females, at least in unadjusted analyses and in middle-aged and elderly healthy individuals [21, 22]. The effect seems to be partially attributed to sex hormones [23] and rather related to a lower prevalence and severity of CVD risk factors. In this regard, the phenotype of diabetic kidney disease differs according to gender, with men more likely to develop A2 or A3 albuminuria [24] and the risk of developing CKD is higher for males at similar blood pressure categories [25]. Different severity and disease characteristics have also been described for primary and systemic glomerulonephritis [26] and for the cardiorenal syndrome [27]. Finally, the phenotype of CKD itself differs in the two sexes; women have been described to have higher serum calcium, phosphorous, and Fibroblast Growth Factor-23 levels than men and possibly a milder anaemia when considering the sex-specific normality ranges [21].

## SEX DISPARITIES IN MORTALITY IN MEN AND WOMEN WITH CKD

In the general population, female life expectancy is longer than male life expectancy and the risk of cardiovascular (CV) events is higher in men than women [28], potentially due to sex hormones [29]. In CKD, the production of sex hormones is altered [30] which may contribute to a lesser protective effect of the female sex on CV events. Most observational studies in CKD cohorts present a higher prevalence of CVD morbidity and mortality in men compared to women.

**Figure 3:**
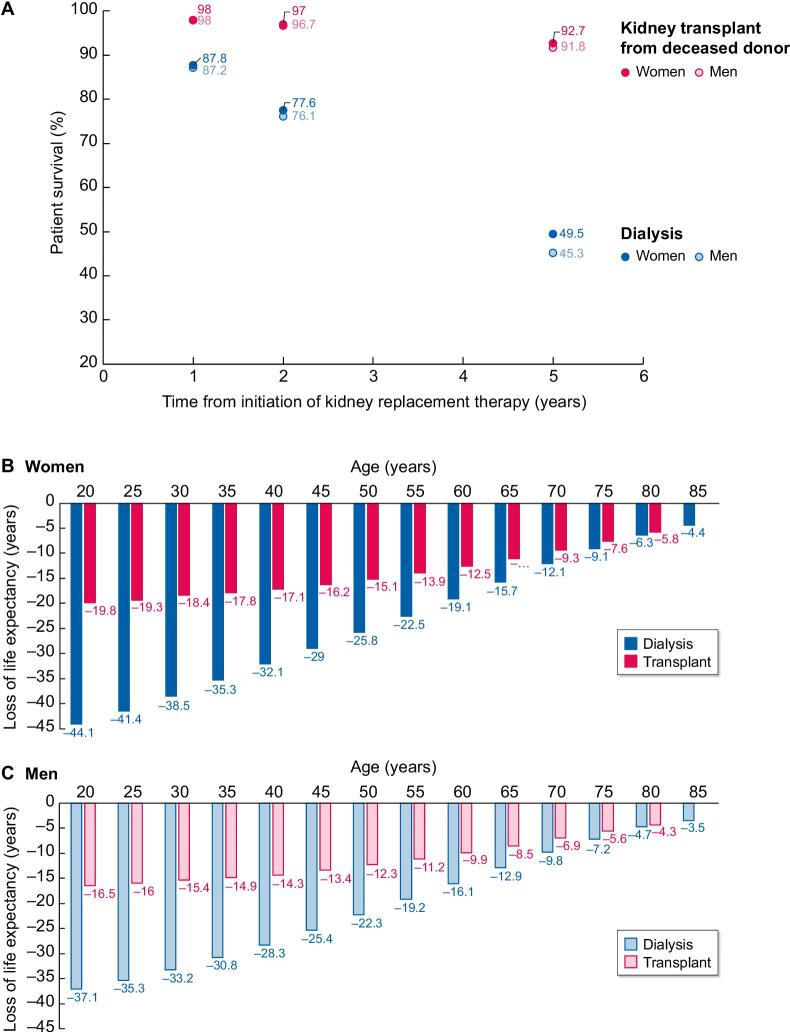
Survival among men and women on kidney replacement therapy (KRT) in Europe according to the ERA registry 2020 report (Ref 18). (**A**) Two and 5-year survival is higher for women on KRT than for men on KRT and the difference is more notable on dialysis. (**B**-**C**) However, women on KRT have a lower life-expectancy compared to the general female population (**B**) than men compared to the male general population (**C**). For the group with the largest absolute loss of life expectancy (those aged 20 to 24 years), the percentual difference in loss of life expectancy between women and men on KRT was 20% and similar for dialysis and transplantation. The number on the age axis refers to the first year of a five-year interval.

However, when comparing mortality in women with CKD to same-age women in the general population and mortality of men with CKD to men in the general population, the CVD burden is higher in women. Dialysis and transplantation registries (like The European Renal Association Registry) can provide such data. While the 2- and 5-year survival rates are higher for European women on KRT than for men on KRT (Fig. 3A), the negative impact of CKD is larger for women with kidney failure compared to the general population of the same sex. The life expectancy of European women on dialysis or kidney transplant recipients is up to 44.8 and 19.8 years less, respectively, than that for same-age women in the general population (Fig. 3B). Men on dialysis or transplantation live 37.1 and 16.5 years less, respectively, compared with men in the general population (Fig. 3C), representing a 21% to 20% difference [18].

CVD outcomes in men and women are discussed in detail separately for non-dialysis CKD patients, patients on dialysis, and kidney transplant recipients.

### CKD population not on dialysis

Few studies introduce sex as a potential confounding factor in prognostic models of CV events in CKD and the odds or hazard ratios (HR) assigned to the sex variable in those studies should not be interpreted as definitive due to potential bias.

In 2013, Hui *et al*. [31] investigated whether age, sex, and race influenced the relationship between eGFR, albuminuria, and CV events in a community-based cohort study with over 11 000 individuals and a follow-up exceeding 11 years. They concluded that CV risk significantly increased with eGFR <70 ml/min/1.73 m^2^, and there was no difference in risk between men and women, although the sample was small and confidence intervals wide. However, high levels of albuminuria had a greater impact on CV risk in women compared to men (1.3- to 1.8-fold higher HR for women at a given albuminuria above 30 mg/g). Furthermore, high albuminuria was significantly associated with CVD only in women.

A meta-analysis conducted in the same year, involving over 2 million participants from general population and CKD cohorts, demonstrated that men had a higher risk of CV mortality than women across all levels of kidney function, although the confidence intervals overlapped for eGFR below 50 ml/min/1.73 m^2^, [32]. Incidentally, the increase in risk with respect to normal eGFR and albuminuria was steeper in women than in men (meaning that although the absolute risk is lower in women, the influence of reduced eGFR and albuminuria is higher).

In 2021, three separate studies from Sweden, Korea, and the USA in CKD populations provided further evidence that men with CKD were more likely to experience CV events than women. Τhe Chronic Renal Insufficiency Cohort (CRIC) study [33] analysed almost 3000 CKD participants followed for nearly 10 years and demonstrated that the adjusted hazard ratios for atherosclerotic events, heart failure, and cardiovascular death were all higher in men than in women. Α Swedish cohort of over 35 000 CKD patients followed for 10 years [34] also found similar results, with men showing a higher cumulative incidence of cardiovascular death. Α smaller cohort of 1780 CKD patients similarly demonstrated that men had a higher likelihood of experiencing adverse cardiovascular events and death compared to women [35].

Recent European and Japanese studies further support these findings. A Japanese study [36] with 5000 patients followed for 10 years observed a higher risk of myocardial infarction in men than in women with CKD. In a pooled analysis of four Italian cohorts of patients with CKD, the risk of CV events was higher in men, although this difference disappeared when systolic blood pressure was over 140 mmHg [37]. Provenzano *et al.* [38] confirmed that male sex was strongly related to the incidence rate of fatal and non-fatal major CV events [HR 1.75, 95% CI 1.18–2.60] in patients with CKD. Finally in a European cohort of G4-G5 CKD patients, not on dialysis and over 65 years old, women had a 18% lower crude risk of first MACE compared to men (HR 0.82, 95% CI 0.69–0.97, *P *= 0.02), but this advantage was lost for women >75 years old and women with diabetes [39] (Table 1).

**Table 1:** Studies comparing CVD outcomes in women and men in non-dialysis CKD populations.

Author, Ref number	Year, type of study, country	Number of patients	Follow-up (years)	CVD outcome definition	CVD outcomes women vs men	All-cause mortality women vs men
Toth-Manikowski S [33]	2021, prospective, longitudinal cohort study (CRIC)-USA	3939	9.6	1) composite outcome (MI, stroke, or peripheral artery disease); 2) incident HF; 3) cardiovascular death	**Composite outcome IR** (/1000person-years) 19 vs 27; HR 0.73 (95% CI: 0.60–0.89) **HF** IR (/1000person-years) 24 vs 27; HR 0.79 (95% CI:0.65–0.95) **Cardiovascular death** IR (/1000person-years) 10 vs 15; HR 0.60 (95% CI: 0.46–0.77)	IR (/1000 person-years) 29 vs 39; HR 0.58 95%CI: 0.49–0.69)
Swartling O [34]	2021, observational cohort study, Sweden	35 000	10	cardiovascular mortality	HR 0.83 [95% CI, 0.76–0.90]	IR (/1000 person-years) 92 vs 106; adjusted HR 0.90 (95% CI 0.85–0.94)
Shiraishi YA [36]	2023, cohort study, Japan	5163	10	stroke, MI, and SD	**Stroke** CI (/100 000 person-years) 239 vs 515; HR 0.87 (0.48–1.51) vs 1.01 (0.56–1.86) **M**I CI (/100 000 person-years) 28 vs 252; HR 2.09 (0.29-15 vs 3.55 (1.25–10.06) **SD** CI (/100 000 person-years) 43 vs 62.5; HR 2.15 (0.41–11.3 vs 0.85 (0.17–4.25)	NA
Borrelli S [37]	2023, pooled analysis of 4 cohorts, Italy	NA	4	composite CV end point (cardiovascular death and non-fatal MI, congestive HF, stroke, revascularization, peripheral vascular disease, non-traumatic amputation)	HR 0.73 (95% CI 0.60–0.89)	NA
Provenzano PF [38]	2023, cohort, south of Italy	759	3	fatal and non-fatal CV events (MI, HF, arrhythmia; stroke; peripheral vascular disease; major arterial or venous thrombotic episodes)	HR (male vs female) 1.78, (95% CI 1.03–3.09)	HR (male vs female) 0.65, (95% CI 0.34–1.25)
Astley ME [39]	2023, prospective, cohort study, Europe (EQUAL study)	1736	3.8	1) MACE (comorbidity or hospitalization due to cerebrovascular disease, MI, peripheral vascular disease, congestive HF, arrythmias, CHD, angina pectoris); 2) death due to MI, HF, cardiac arrest, cerebrovascular accident	First MACE IR (/1000 person-years) 23 vs 27; unadjusted HR 0.82 (0.69–0.97, *P* = 0.02) Fatal MACE IR (/1000 person-years) 3 vs 4; unadjusted 0.84 (0.61–1.16, *P* = 0.30)	NA

CHD, coronary heart disease; MI, myocardial infarction; SD, sudden death; HF, heart failure; IR, incidence rate; CI, crude incidence; HR, hazard ratio; MACE, major acute CV events; NA, not available.

### Dialysis patients

Women on dialysis seem to have lost most of the survival advantage over men in dialysis in most observational studies. But as indicated above, mortality is higher in women on dialysis compared to women in the general population, than when the same comparison is done for men [18].

Among 35 964 participants from 12 countries in Dialysis Outcomes and Practice Patterns Study (DOPPS), mortality was similar in men and women on dialysis in all age groups in all DOPPS countries, except Japan, while in the general population male-to-female mortality rate ratios varied from 1.5 to 2.6. Certain haemodialysis characteristics showed a significant sex interaction with mortality: hemodialysis catheter use displayed the largest difference in mortality risk between men (HR = 1.11 in comparison to no catheter use) and women (HR = 1.33 in comparison to no catheter use), interaction *P* = 0.001 [40]. In the Australian and New Zealand Dialysis and Transplant Registry (ANZDATA), excess all-cause mortality was 7% higher in women on haemodialysis than in men (adjusted excess mortality ratio 1.07, 1.04 –1.10, *P *< 0.001) while in peritoneal dialysis the sex difference in excess mortality varied by age: female patients aged 30–49 years had 15% lower excess mortality than males (0.85, 0.76–0.95, *P *= 0.004) while female aged ≥75 years had 24% excess mortality (1.24, 1.11–1.38, *P *< 0.001) compared with male patients. Although the proportion of CV deaths was higher in male than in female patients (4.7% higher, 3.9%–5.6%, *P *< 0.001), cardiovascular mortality was higher in women on dialysis compared to women in the general population than when the same comparison was done for men (standardised mortality ratio 8.7 (8.4–9.0), and 5.7 (5.6–5.9) for females and males, respectively) [41], a remark in agreement with European Registry data [18].

In a Japanese study [42], women on dialysis had a lower risk of all-cause death than men (19.9% vs 28.6%, *P *< 0.001 and HR: 0.70, 95% CI 0.54–0.90), with no differences in CV death (8.6 vs 10.9%, *P *= 0.177). In patients without CVD, female sex was a strong independent protective risk factor for all-cause mortality (HR 0.46, 95% CI: 0.30–0.70) while this advantage was lost for patients with CVD (HR 0.92, 95% CI: 0.67–1.24). In another study, women in dialysis had higher adjusted rates for the composite outcome of CV hospitalization or all-cause death overall than men (HR 2.5; 95%CI 1.1–5.6; *P *= 0.03) [43].

In a large cohort of 108 963 Europeans on dialysis during a 5-year follow-up, young women (under 45 years of age) had higher non-cardiovascular mortality risk than men, mainly due to infections, which is opposite to trends observed in the general population. In other age categories (>45 years), women had lower CV mortality [44]. In all age categories, diabetic women had an increased risk of all-cause death compared with men, an effect mainly attributed to non-cardiovascular deaths.

In a systematic review and meta-analysis of 23 studies examining 86 915 patients on haemodialysis, sex (women versus men) did not significantly affect all-cause mortality but did have a negative effect on cardiac death (RR: 1.41; 95% CI: 1.11–1.80; *P *= 0.005) [45]. In a recent meta-analysis including 48 studies with 99 822 participants (51 069 men, 48 753 women) and combining reported and calculated risk estimates, males had higher cardiovascular mortality among CKD patients than women (risk estimate 1.13, 95%CI 1.03–1.25) [46].

### Kidney transplant recipients

Epidemiological information on CVD in kidney transplant recipients (KTRs) has relied mainly on registry databases and retrospective studies, whereas only recently prospective studies have been added [47–50]. Although CV risk is reduced following kidney transplantation compared with dialysis, the incidence of CVD in KTRs is three to five times higher than in the general population [47, 48] and CVD remains the principal cause of death, accounting for 20–35% of overall mortality [50–52].

Available data suggest gender disparities in transplant access with men in the USA having greater access to transplantation whereas differences regarding transplant outcomes remain inconclusive [12]. There is scarce evidence on gender differences with respect to CV events and outcomes in KTRs. Previous studies identified male sex as an independent variable for CV events prediction in KTRs [53, 54]. Among 30 325 KTRs in England, men had a 20% higher risk than women for non-fatal MACE defined as any hospital admission with myocardial infarction, stroke, unstable angina, heart failure, any coronary revascularization procedure within 12 months of transplant surgery [55]. However, among 16 329 KTRs in the Australian and New Zealand registry, the standardized mortality rates were higher among transplanted women across all age groups than among men compared with the general population, despite male sex being an independent risk factor for cardiac death posttransplant [56]. Likewise, results from a meta-analysis across three transplant registries showed higher excess all-cause mortality risks in female than male KTRs compared to the same sex in the general population at all ages, except 45 to 59 years [57]. However, sex differences in excess mortality were statistically significant only when the donor was male [57].

Finally, only 33.7% of participants in 24 KTR trials were women, suggesting underrepresentation of women in kidney transplantation trials, including ones examining cardiometabolic risk [58]. A more balanced representation of women in these trials will contribute to further exploring and understanding of gender disparities in posttransplant care.

## RISK FACTORS FOR CVD IN MEN AND WOMEN WITH CKD

Men and women share traditional CV risk factors (hypertension, hyperlipidaemia, diabetes, smoking), but their prevalence and impact on CVD vary by sex [59]. Prevalence of hypertension is higher among men compared to women with a steeper increase in menopausal females [60]. This could be attributed to the impact of oestrogens on renin angiotensin system and immune cell activation, endothelin and sympathetic nervous system function and antihypertensive pharmacokinetics [61–63].

Office blood pressure (BP) levels in the CKD population show no consistent gender differences and seem not to modify CKD progression [21, 64, 65]. However, in a Chinese study, men were more sensitive to hypertension-associated GFR decline [66], while African-American men with early CKD had poorer hypertension control than women [67]. On the other hand, ambulatory BP assessments disclose the high prevalence of ‘white coat’ and ‘masked’ hypertension in CKD populations [68], and demonstrate stronger associations with CKD progression, CVD morbidity and mortality (particularly nighttime BP or short-term BP variability) compared with office BP [69, 70]. Sex differences do exist in ambulatory BP measurements in dialysis and transplantation patients [71, 72]; men with CKD (stages 2–5) have higher daytime and nighttime systolic BP than women, which may be the key contributor to male higher risk of adverse outcomes [73].

Women with CKD seem to have less likely metabolic syndrome and diabetes [74], while female patients with diabetic kidney disease present milder albuminuria and a better response to therapy than men [75]. Smoking and diabetes increase more the CVD risk in women compared to men in the general population [76, 77], and a similar pattern is seen in the CKD population [78, 79], while data about the impact of sex on kidney outcomes in diabetes are contradictory [80, 81]. Pre-eclampsia and hypertension during pregnancy are important risk factors for CKD incidence and increased long-term CVD risk for both the mothers and their offspring [82–84].

Non-traditional CVD risk factors in CKD like vascular calcification and inflammation [59] may have different sex phenotypes too. Women seem to present higher levels of platelet aggregation and lower response to aspirin [85], while pulse pressure and arterial stiffness increase more with aging in women than in men [86, 87]. Systemic inflammation in CKD is common [88] and increased serum inflammatory biomarkers like C-reactive protein (CRP), inter-leukin-6 (IL-6) and tumour necrosis factor (TNF) are associated with CVD and all-cause mortality [89]. We do not have direct data about sex differences in systemic inflammation. However, women have higher autoimmunity risk as exemplified by the higher incidence of systemic lupus erythematosus and rheumatoid arthritis [74, 82]. Among dialysis patients, coronary artery calcification had a sex-specific signature, as females were more often inflamed (higher IL-6 and TNF levels) than men [90]. Finally, sex differences in CVD risk factors do influence outcomes as adjustment for traditional CV risk factors and CRP in observational studies in non-dialysis CKD populations, reduced the sex risk difference for heart failure, death [33], or MACE [39].

## POTENTIAL REASONS FOR SEX DISPARITIES

Biological and non-biological factors may contribute to sex disparities in CVD among patients with CKD.

### Biological factors

Genetic and metabolic differences between males and females may account for different disease susceptibility and response to therapy [91] (Table 2). Males and females differ genetically as male cells have a Y chromosome and a single X chromosome, while each female cell expresses one of two available X chromosomes. As a result, urogenital development differs, different gonads and sex hormones are generated, and different embryonic structures disappear or evolve. During adult life, the hormonal environment also differs (androgens predominate in males and oestrogens in females) and physiological changes may create further differences: iron deficiency is more common in females, pregnancy leads to transient hormonal and kidney function changes and to exposure of foreign antigens that may sensitize to future kidney grafts, and menopause leads to a relatively abrupt loss of oestrogens. These biological differences result in physiological differences between males and females: autoimmunity (e.g. lupus nephritis) and urinary tract infection are more common in women, while X-linked genetic diseases are generally more severe in males and sex is a genetic modifier of the pharmacological response to drugs [62].

**Table 2:** Main biological differences between males and females that may modify susceptibility to kidney and cardiovascular disease.


• **Primary drivers of biological differences**
○ Sex-specific gene expression: X and Y chromosomes and genetic imprinting of autosomes
○ Sex hormone-dependent changes
▪ Androgen surge during development
• Permanent differences in organ and tissue structure
• Epigenetic regulation of gene expression
▪ Persistent sex hormone differences from puberty and throughout life, female menopause
• **Biological differences secondary to primary drivers**
○ Impact of menses: iron deficiency
○ Impact of pregnancy: large, reversible changes in kidney function, potential sensitization to foreign antigens
○ Impact of gender differences in behaviour and lifestyles on biological variables
○ Different energy metabolism
○ Different disease susceptibility, e.g. in women
▪ Increased susceptibility to autoimmunity (e.g. lupus nephritis) and urinary tract infection
▪ Increased susceptibility to heart failure with preserved ejection fraction
○ Different response to therapeutic interventions
○ Different resilience to specific forms of cell death (e.g. ferroptosis)
○ Different interaction with the gut microbiota

Both primary drivers of biological differences and some examples of biological consequence of these primary drivers are shown. Molecular mechanisms have been well characterized in mice, but the clinical relevance of many of the findings remains unclear. Conversely, the molecular basis of some epidemiological differences observed in humans remain poorly characterized.

**Figure 4:**
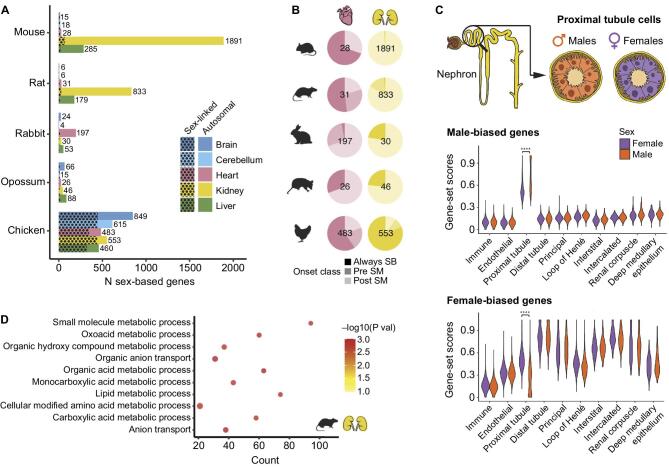
Sex-biased gene expression in the kidney. (**A**) Number of sex-biased genes by species and organ. Spotted pattern indicates genes located on sex chromosomes. **(B**) Percentage of sex-biased genes belonging to each of the onset classes: always sex-biased (Always SB), sex-biased pre–sexual maturity (Pre SM), or sex-biased post–sexual maturity (Post SM). Shown is the total number of sex-biased genes per organ and species inside each pie plot. (**C**) Distribution of male-biased (up) and female-biased (down) gene-set scores according to cell type and separated by male and female cells in adult mouse kidney scRNA-seq dataset (data from [92]) (**** adjusted *P* < 0.0001). (**D**) Enriched biological processes among genes that become sex biased after sexual maturity in rat kidney (*n* = 688; adjusted *P* < 0.05). Reproduced with permission from Ref 92.

In preclinical studies, kidneys were among organs with high levels of sex-biased expression, proximal tubular cells having the highest sexual dimorphism [92] (Fig. 4). These differences only appeared around the time of sexual maturity. The heart and vessels also displayed some degree of sex-biased gene expression, shared by humans [92, 93]. These differences relate mainly to metabolism genes, may underlie differences in energy metabolism between sexes that regulate predisposition to kidney disease and in proximal tubules are driven by both sex hormone receptor transcription factors (e.g. the androgen receptor, Ar) and other sex-biased transcription factors (e.g. Hnf4a in males and Ap-2 in females) [92–94].

These findings may explain more subtle differences. Regulated necrosis by ferroptosis has emerged as the key contributor to both AKI and CKD [95, 96]. Ferroptosis is iron-dependent and relative iron deficiency may be protective (as is the case of females during their reproductive phase). Additionally, gene expression differences make female proximal tubules resistant to ferroptosis, and this may underlie sexual dimorphism in kidney injury and repair, at least in mice [97]. In another example, gut microbiota results in sex-specific diurnal rhythms of gene expression and metabolism in mice that may influence kidney disease and CVD [98]. However, despite extensive mouse studies, human information on sex-related molecular mechanisms in kidney disease and CVD remains scarce [8].

### Non-biological factors

Overall, very little research has been done examining the role of sex in CVD in patients with CKD [2]. However, information on the role of gender on overall health may apply to CVD in the general population and in patients with CKD.

Globally, multiple gender inequalities impact on health-related outcomes. More women than men are likely to live in extreme poverty, live with food insecurity, experience domestic violence, have limited access to secondary education or healthcare, engage in underpaid or unpaid work, be victims of trafficking, while fewer women hold leadership positions or are researchers [20, 99, 100]. This has been attributed to lack of economic power, social position, cultural norms, and competing responsibilities [99, 101]. Women are also less likely to receive evidence-based treatments than men even in high-income countries, especially if being treated by a male physician [91].

Measures of gender, more commonly associated with women including child care, social support, personality traits, and education level are associated with worse cardiovascular outcomes [102, 103]. Various presenting symptoms of acute coronary syndrome differ between sexes (women refer more often with nausea, back and neck pain) [104] and diagnosis of heart disease tends to be more delayed in women [105]. Women are also less likely to receive evidence-based management for myocardial infarction [91]. Finally, elderly women are more likely to choose conservative care and report higher symptom burden and severity than men on dialysis [8].

By contrast, male gender is also associated with some disadvantages [106]. More men than women are at higher risk of injury, homicide, occupational exposures, poisoning and either have less access or are less likely to use screening and prevention programs, or engage with primary care [20, 106]. In a recent clinical trial of screening methods for albuminuria, participation of men was 6%–9% lower and acceptance of a full evaluation after testing positive was also an additional 4%–8% lower than in women, thus decreasing the opportunity for early identification of CKD or high CVD risk [107] Depression is diagnosed less often in men and men are twice as likely to commit suicide than women [108]. Men are more likely to adopt avoidance behaviours such as smoking and drinking rather than dealing with illness [108] and generally have poorer adherence to long-term medication including antihypertensive treatments [108]. In pre-dialysis CKD, men tend to have worse adherence to medication, diet and healthcare-seeking behaviour, which may contribute to faster GFR decline [103, 109]. Moreover, after starting dialysis, they are more likely to continue smoking and drinking alcohol [108, 110].

Overall, gender plays a significant role in shaping individuals’ choices and attitudes towards health. This underscores the importance of gaining a deeper understanding of these factors and implementing gender-specific corrective actions.

## IMPROVING OUTCOMES FOR WOMEN WITH CKD AND CVD

Health results in CVD and CKD can be improved through a gender-based approach built on the following premises:

• Characterization of gender-specific CV and renal risk factors. Gender-specific risk factors may differ because of different lifestyles or sex differences in pathophysiology. In some studies, men had a higher burden of hypertension, obesity, or dietary sodium than females [75, 111]. Smoking appears to be a risk factor for hyperkalemia only in men [112].
• Definition of gender-specific biomarkers and thresholds. The range of biomarker normal values and cut-off values for risk stratification may differ for men and women and the optimal values for women should be defined. KDIGO cut-off values for albuminuria are similar in men and women (e.g. UACR 30 mg/g or albuminuria 30 mg/day) but the creatinine denominator differs for men and women. In this regard, in a nephrology clinic-based DKD series, albuminuria better predicted worsening eGFR in men than in women [75].
• Avoiding gender biases in the diagnosis and treatment of CVD in CKD patients and ensuring that all patients receive evidence-based care regardless of gender. Only 40% of patients on KRT worldwide are women, despite reports that there are more women than men with CKD in earlier stages [113]. Financial and social disadvantages may bias against access of women to diagnostic and therapeutic interventions and RT, mostly in undeveloped countries [114, 115].
• Evidence-based therapies. This requires a separate analysis of men and women outcomes in randomized clinical trials (RCT) and facilitating access of women to RCTs. Women are clearly under-represented in most CKD RCTs; from 1995 to 2022, in 192 RCTs, women represented 66 875 (45%) of 147 136 participants. Several reasons may contribute to this underrepresentation: in some conditions, males may have more severe disease that meets entry criteria. However, it should not be the result of social or reproductive biases and should not cause trials to be underpowered for women. In 39 of those trials, there were differences in efficacy between genders, but no differences in safety issues were demonstrated [116].
• Finally, evidence is needed on the accuracy of biomarkers and the efficacy and safety of interventions in the trans community [117].

## CONCLUSIONS

While the absolute prevalence of CVD is less in women than in men with CKD, excess mortality compared to same-sex general population is higher in women than in men on KRT. Kidney transplantation lowers the cardiovascular risk of patients compared to those who remain on dialysis, but life expectancy in transplanted women compared to the general population is still 20% shorter than in transplanted men compared to same-age men in general population. Traditional CV risk factors like hypertension and diabetes present sex differences in prevalence and pregnancy complications like pre-eclampsia are associated with a strong, long-term CV risk in female lives. Hormones and differences of gene expression may explain CVD sex disparities in CKD. Moreover cultural, social, geographical, and financial factors impact women's late referral, delayed therapy, and under-representation in trials, while male sex is marred by less compliance and health-seeking behaviour.

Awareness of sex disparities in CVD in CKD populations is the first step of the process to diagnose and treat patients according to gender. Unravelling sex and gender differences in pathogenetic mechanisms may contribute to develop specific diagnostic tools and optimize targeted treatment protocols, which can really improve CV health of men and women with CKD. Therefore, studies focusing on the role of sex and gender on CVD outcomes and CKD progression are warranted. Finally, as biological factors involved in sex disparities are not expected to change without pharmacological intervention, efforts should focus on the elimination of societal and cultural factor,s which hinder both sexes from comprehensive nephrology care.

## Data Availability

No new data were generated or analysed in support of this research.

## References

[1] Crews DC, Liu Y, Boulware LE. Disparities in the burden, outcomes, and care of chronic kidney disease. Curr Opin Nephrol Hypertens 2014;23:298–305. 10.1097/01.mnh.0000444822.25991.f6 24662984 PMC4126677

[2] Yi TW, Levin A. Sex, gender, and cardiovascular disease in chronic kidney disease. Semin Nephrol 2022;42:197–207. 10.1016/j.semnephrol.2022.04.009 35718366

[3] den Ruijter HM, Haitjema S, Asselbergs FW et al. Sex matters to the heart: a special issue dedicated to the impact of sex related differences of cardiovascular diseases. Atherosclerosis 2015;241:205–7. 10.1016/j.atherosclerosis.2015.05.003 26003338

[4] Chesnaye NC, Carrero JJ, Hecking M et al. Differences in the epidemiology, management and outcomes of kidney disease in men and women. Nat Rev Nephrol 2024;20:7–20. 10.1038/s41581-023-00784-z 37985869

[5] Collaborators GBDCoD. Global, regional, and national age-sex-specific mortality for 282 causes of death in 195 countries and territories, 1980-2017: a systematic analysis for the Global Burden of Disease Study 2017. Lancet 2018;392:1736–88. 10.1016/S0140-6736(18)32203-7 30496103 PMC6227606

[6] Jha V, Wang AY, Wang H. The impact of CKD identification in large countries: the burden of illness. Nephrol Dial Transplant 2012;27 Suppl 3:iii32–8. 10.1093/ndt/gfs113 23115140

[7] Heron M. Deaths: leading causes for 2018. Natl Vital Stat Rep 2021;70:1–115. 34029179

[8] Carrero JJ, Hecking M, Chesnaye NC et al. Sex and gender disparities in the epidemiology and outcomes of chronic kidney disease. Nat Rev Nephrol 2018;14:151–64. 10.1038/nrneph.2017.181 29355169

[9] Neugarten J, Acharya A, Silbiger SR. Effect of gender on the progression of nondiabetic renal disease: a meta-analysis. J Am Soc Nephrol 2000;11:319–29. 10.1681/ASN.V112319 10665939

[10] Hecking M, Tu C, Zee J et al. Sex-specific differences in mortality and incident dialysis in the chronic kidney disease outcomes and practice patterns study. Kidney Int Rep 2022;7:410–23. 10.1016/j.ekir.2021.11.018 35257054 PMC8897674

[11] Antlanger M, Noordzij M, van de Luijtgaarden M et al. Sex differences in Kidney replacement therapy initiation and maintenance. Clin J Am Soc Nephrol 2019;14:1616–25. 10.2215/CJN.04400419 31649071 PMC6832047

[12] Katz-Greenberg G, Shah S. Sex and gender differences in kidney transplantation. Semin Nephrol 2022;42:219–29. 10.1016/j.semnephrol.2022.04.011 35718368 PMC10065984

[13] Johansen KL, Chertow GM, Gilbertson DT et al. US Renal Data System 2022 Annual Data Report: epidemiology of kidney disease in the United States. Am J Kidney Dis 2023;81:A8–A11. 10.1053/j.ajkd.2022.12.001 36822739 PMC10807034

[14] Mills KT, Xu Y, Zhang W et al. A systematic analysis of worldwide population-based data on the global burden of chronic kidney disease in 2010. Kidney Int 2015;88:950–7. 10.1038/ki.2015.230 26221752 PMC4653075

[15] Collaboration GBDCKD. Global, regional, and national burden of chronic kidney disease, 1990-2017: a systematic analysis for the Global Burden of Disease Study 2017. Lancet 2020;395:709–33. 10.1016/S0140-6736(20)30045-3 32061315 PMC7049905

[16] Florea A, Jacobs ET, Harris RB et al. Chronic kidney disease unawareness and determinants using 1999-2014 National Health and Nutrition Examination Survey Data. J Public Health (Oxf) 2022;44:532–40. 10.1093/pubmed/fdab112 33837421 PMC9618171

[17] Chang HJ, Lin KR, Lin MT et al. Associations between lifestyle factors and reduced kidney function in US older adults: NHANES 1999-2016. Int J Public Health 2021;66:1603966. 10.3389/ijph.2021.1603966 34335140 PMC8319092

[18] Astley ME, Boenink R, Abd ElHafeez S et al. The ERA Registry Annual Report 2020: a summary. Clin Kidney J 2023;16:1330–54. 10.1093/ckj/sfad087 37529647 PMC10387405

[19] Gifford FJ, Methven S, Boag DE et al. Chronic kidney disease prevalence and secular trends in a UK population: the impact of MDRD and CKD-EPI formulae. QJM 2011;104:1045–53. 10.1093/qjmed/hcr122 21821654

[20] Garcia GG, Iyengar A, Kaze F et al. Sex and gender differences in chronic kidney disease and access to care around the globe. Semin Nephrol 2022;42:101–13. 10.1016/j.semnephrol.2022.04.001 35718358

[21] Ricardo AC, Yang W, Sha D et al. Sex-related disparities in CKD progression. J Am Soc Nephrol 2019;30:137–46. 10.1681/ASN.2018030296 30510134 PMC6317604

[22] Melsom T, Norvik JV, Enoksen IT et al. Sex differences in age-related loss of kidney function. J Am Soc Nephrol 2022;33:1891–902. 10.1681/ASN.2022030323 35977806 PMC9528336

[23] Kang SC, Jhee JH, Joo YS et al. Association of reproductive lifespan duration and chronic kidney disease in postmenopausal women. Mayo Clin Proc 2020;95:2621–32. 10.1016/j.mayocp.2020.02.034 33168161

[24] Yu MK, Lyles CR, Bent-Shaw LA et al. Risk factor, age and sex differences in chronic kidney disease prevalence in a diabetic cohort: the pathways study. Am J Nephrol 2012;36:245–51. 10.1159/000342210 22964976 PMC3510352

[25] Satoh M, Hirose T, Nakayama S et al. Blood pressure and chronic kidney disease stratified by gender and the use of antihypertensive drugs. J Am Heart Assoc 2020;9:e015592. 10.1161/JAHA.119.015592 32794421 PMC7660816

[26] Beckwith H, Lightstone L, McAdoo S. Sex and gender in glomerular disease. Semin Nephrol 2022;42:185–96. 10.1016/j.semnephrol.2022.04.008 35718365

[27] Cobo Marcos M, de la Espriella R, Gayan Ordas J et al. Sex differences in cardiorenal syndrome: insights from CARDIOREN Registry. Curr Heart Fail Rep 2023;20:157–67. 10.1007/s11897-023-00598-x 37222949

[28] Roth GA, Mensah GA, Johnson CO et al. Global burden of cardiovascular diseases and Risk factors, 1990-2019: update from the GBD 2019 study. J Am Coll Cardiol 2020;76:2982–3021. 10.1016/j.jacc.2020.11.010 33309175 PMC7755038

[29] Kaur H, Werstuck GH. The effect of testosterone on cardiovascular disease and cardiovascular risk factors in men: a review of clinical and preclinical data. CJC Open 2021;3:1238–48. 10.1016/j.cjco.2021.05.007 34888506 PMC8636244

[30] Valdivielso JM, Jacobs-Cacha C, Soler MJ. Sex hormones and their influence on chronic kidney disease. Curr Opin Nephrol Hypertens 2019;28:1–9. 10.1097/MNH.0000000000000463 30320621

[31] Hui X, Matsushita K, Sang Y et al. CKD and cardiovascular disease in the Atherosclerosis Risk in Communities (ARIC) study: interactions with age, sex, and race. Am J Kidney Dis 2013;62:691–702. 10.1053/j.ajkd.2013.04.010 23769137 PMC3783539

[32] Nitsch D, Grams M, Sang Y et al. Associations of estimated glomerular filtration rate and albuminuria with mortality and renal failure by sex: a meta-analysis. BMJ 2013;346:f324. 10.1136/bmj.f324 23360717 PMC3558410

[33] Toth-Manikowski SM, Yang W, Appel L et al. Sex differences in cardiovascular outcomes in CKD: findings from the CRIC study. Am J Kidney Dis 2021;78:200–209 e1. 10.1053/j.ajkd.2021.01.020 33857532 PMC8316276

[34] Swartling O, Rydell H, Stendahl M et al. CKD progression and mortality among men and women: a nationwide study in Sweden. Am J Kidney Dis 2021;78:190–9 e1. 10.1053/j.ajkd.2020.11.026 33434591

[35] Jung CY, Heo GY, Park JT et al. Sex disparities and adverse cardiovascular and kidney outcomes in patients with chronic kidney disease: results from the KNOW-CKD. Clin Res Cardiol 2021;110:1116–27. 10.1007/s00392-021-01872-5 34003323

[36] Shiraishi YA, Ishikawa Y, Ishikawa J et al. Age and sex differences in the risk of cardiovascular diseases by chronic kidney disease in a general Japanese population. Heart Vessels 2023;38:1164–71. 10.1007/s00380-023-02264-7 37039880

[37] Borrelli S, Garofalo C, Gabbai FB et al. Sex difference in cardiovascular risk in patients with chronic kidney disease: pooled analysis of four cohort studies. Nephrol Dial Transplant 2023:gfad036. 10.1093/ndt/gfad036. Online ahead of print. 36796825

[38] Provenzano PF, Caridi G, Parlongo G et al. Are there sex differences in cardiovascular outcomes in non-dialysis CKD patients? Clin Kidney J 2023;16:2141–6. 10.1093/ckj/sfad174 37915890 PMC10616483

[39] Astley M, Caskey FJ, Evans M et al. The impact of gender on the risk of cardiovascular events in older adults with advanced chronic kidney disease. Clin Kidney J 2023;16:2396–404. 10.1093/ckj/sfad088 38046000 PMC10689190

[40] Hecking M, Bieber BA, Ethier J et al. Sex-specific differences in hemodialysis prevalence and practices and the male-to-female mortality rate: the Dialysis Outcomes and Practice Patterns Study (DOPPS). PLoS Med 2014;11:e1001750. 10.1371/journal.pmed.1001750 25350533 PMC4211675

[41] De La Mata NL, Rosales B, MacLeod G et al. Sex differences in mortality among binational cohort of people with chronic kidney disease: population based data linkage study. BMJ 2021;375:e068247. 10.1136/BMJ-2021-068247 34785509 PMC8593820

[42] Kozaki Y, Morinaga T, Fukatsu A et al. Sex differences in clinical outcomes in Japanese incident dialysis patients: a prospective observational multicenter study. Clin Exp Nephrol 2022;26:466–75. 10.1007/s10157-021-02168-8 35048329

[43] Guajardo I, Ayer A, Johnson AD et al. Sex differences in vascular dysfunction and cardiovascular outcomes: the cardiac, endothelial function, and arterial stiffness in ESRD (CERES) study. Hemodial Int 2018;22:93–102. 10.1111/hdi.12544 28272770

[44] Carrero JJ, de Jager DJ, Verduijn M et al. Cardiovascular and noncardiovascular mortality among men and women starting dialysis. Clin J Am Soc Nephrol 2011;6:1722–30. 10.2215/CJN.11331210 21734088

[45] Ma L, Zhao S. Risk factors for mortality in patients undergoing hemodialysis: a systematic review and meta-analysis. Int J Cardiol 2017;238:151–8. 10.1016/j.ijcard.2017.02.095 28341375

[46] Shajahan S, Amin J, Phillips JK et al. Relationship between sex and cardiovascular mortality in chronic kidney disease: a systematic review and meta-analysis. PLoS One 2021;16:e0254554. 10.1371/journal.pone.0254554 34252153 PMC8274915

[47] Methven S, Steenkamp R, Fraser S. UK Renal Registry 19th Annual Report: chapter 5 survival and causes of death in UK adult patients on Renal Replacement Therapy in 2015: national and centre-specific analyses. Nephron 2017;137 Suppl 1:117–50. 10.1159/000481367 28930724

[48] Saran R, Robinson B, Abbott KC et al. US Renal Data System 2018 Annual Data Report: epidemiology of kidney disease in the United States. Am J Kidney Dis 2019;73:A7–A8. 10.1053/j.ajkd.2019.01.001 30798791 PMC6620109

[49] Ribic CM, Holland D, Howell J et al. Study of cardiovascular outcomes in renal transplantation: a prospective, multicenter study to determine the incidence of cardiovascular events in renal transplant recipients in Ontario. Can J Kidney Health Dis 2017;4. 10.1177/2054358117713729 PMC5476328 28660072

[50] Kim JE, Park J, Park S et al. De novo major cardiovascular events in kidney transplant recipients: a comparative matched cohort study. Nephrol Dial Transplant 2023;38:499–506. 10.1093/ndt/gfac144 35396847

[51] Meier-Kriesche HU, Schold JD, Srinivas TR et al. Kidney transplantation halts cardiovascular disease progression in patients with end-stage renal disease. Am J Transplant 2004;4:1662–8. 10.1111/j.1600-6143.2004.00573.x 15367222

[52] Gillis KA, Patel RK, Jardine AG. Cardiovascular complications after transplantation: treatment options in solid organ recipients. Transplant Rev (Orlando) 2014;28:47–55. 10.1016/j.trre.2013.12.001 24412041

[53] Israni AK, Snyder JJ, Skeans MA et al. Predicting coronary heart disease after kidney transplantation: patient outcomes in renal transplantation (PORT) study. Am J Transplant 2010;10:338–53. 10.1111/j.1600-6143.2009.02949.x 20415903

[54] Seoane-Pillado MT, Pita-Fernandez S, Valdes-Canedo F et al. Incidence of cardiovascular events and associated risk factors in kidney transplant patients: a competing risks survival analysis. BMC Cardiovasc Disord 2017;17:72. 10.1186/s12872-017-0505-6 28270107 PMC5341360

[55] Anderson B, Qasim M, Evison F et al. A population cohort analysis of English transplant centers indicates major adverse cardiovascular events after kidney transplantation. Kidney Int 2022;102:876–84. 10.1016/j.kint.2022.05.017 35716956

[56] Wyld MLR, De La Mata NL, Masson P et al. Cardiac mortality in kidney transplant patients: a population-based cohort study 1988-2013 in Australia and New Zealand. Transplantation 2021;105:413–22. 10.1097/TP.0000000000003224 32168042

[57] Vinson AJ, Zhang X, Dahhou M et al. A multinational cohort study uncovered sex differences in excess mortality after kidney transplant. Kidney Int 2023;103:1131–43. 10.1016/j.kint.2023.01.022 36805451

[58] Vinson AJ, Ahmed SB. Representation of women in contemporary kidney transplant trials. Transpl Int 2023;36:11206. 10.3389/ti.2023.11206 37125385 PMC10141646

[59] Jankowski J, Floege J, Fliser D et al. Cardiovascular disease in chronic kidney disease: pathophysiological insights and therapeutic options. Circulation 2021;143:1157–72. 10.1161/CIRCULATIONAHA.120.050686 33720773 PMC7969169

[60] Ramirez LA, Sullivan JC. Sex differences in hypertension: where we have been and where we are going. Am J Hypertens 2018;31:1247–54. 10.1093/ajh/hpy148 30299518 PMC6233684

[61] Sarafidis P, Burnier M. Sex differences in the progression of kidney injury and risk of death in CKD patients: is different ambulatory blood pressure control the underlying cause? Nephrol Dial Transplant 2021;36:1965–7. 10.1093/ndt/gfab115 33848343

[62] Mauvais-Jarvis F, Berthold HK, Campesi I et al. Sex- and gender-based pharmacological response to drugs. Pharmacol Rev 2021;73:730–62. 10.1124/pharmrev.120.000206 33653873 PMC7938661

[63] Song JJ, Ma Z, Wang J et al. Gender differences in hypertension. J Cardiovasc Transl Res 2020;13:47–54. 10.1007/s12265-019-09888-z 31044374

[64] Minutolo R, Gabbai FB, Chiodini P et al. Sex differences in the progression of CKD among older patients: pooled analysis of 4 cohort studies. Am J Kidney Dis 2020;75:30–38. 10.1053/j.ajkd.2019.05.019 31409508

[65] Chesnaye NC, Dekker FW, Evans M et al. Renal function decline in older men and women with advanced chronic kidney disease-results from the EQUAL study. Nephrol Dial Transplant 2021;36:1656–63. 10.1093/ndt/gfaa095 32591814 PMC8396396

[66] Wang Q, Xie D, Xu X et al. Blood pressure and renal function decline: a 7-year prospective cohort study in middle-aged rural Chinese men and women. J Hypertens 2015;33:136–43. 10.1097/HJH.0000000000000360 25255396

[67] Duru OK, Li S, Jurkovitz C et al. Race and sex differences in hypertension control in CKD: results from the Kidney Early Evaluation Program (KEEP). Am J Kidney Dis 2008;51:192–8. 10.1053/j.ajkd.2007.09.023 18215697 PMC2866650

[68] Gorostidi M, Sarafidis PA, de la Sierra A et al. Differences between office and 24-hour blood pressure control in hypertensive patients with CKD: a 5,693-patient cross-sectional analysis from Spain. Am J Kidney Dis 2013;62:285–94. 10.1053/j.ajkd.2013.03.025 23689071

[69] Ruiz-Hurtado G, Ruilope LM, de la Sierra A et al. Association between high and very high albuminuria and nighttime blood pressure: influence of diabetes and chronic kidney disease. Diabetes Care 2016;39:1729–37. 10.2337/dc16-0748 27515965

[70] Mallamaci F, Tripepi G, D'Arrigo G et al. Blood pressure variability, mortality, and cardiovascular outcomes in CKD patients. Clin J Am Soc Nephrol 2019;14:233–40. 10.2215/CJN.04030318 30602461 PMC6390905

[71] Korogiannou M, Sarafidis P, Theodorakopoulou MP et al. Sex differences in ambulatory blood pressure levels, control, and phenotypes of hypertension in kidney transplant recipients. J Hypertens 2022;40:356–63. 10.1097/HJH.0000000000003019 34581304

[72] Theodorakopoulou MP, Karagiannidis AG, Alexandrou ME et al. Sex differences in ambulatory blood pressure levels, control and phenotypes of hypertension in hemodialysis patients. J Hypertens 2022;40:1735–43. 10.1097/HJH.0000000000003207 35788097

[73] Minutolo R, Gabbai FB, Agarwal R et al. Sex difference in ambulatory blood pressure control associates with risk of ESKD and death in CKD patients receiving stable nephrology care. Nephrol Dial Transplant 2021;36:2000–7. 10.1093/ndt/gfab017 33693796

[74] Halbesma N, Brantsma AH, Bakker SJ et al. Gender differences in predictors of the decline of renal function in the general population. Kidney Int 2008;74:505–12. 10.1038/ki.2008.200 18496511

[75] Fernandez-Fernandez B, Mahillo I, Sanchez-Rodriguez J et al. Gender, albuminuria and chronic kidney disease progression in treated diabetic kidney disease. J Clin Med 2020;9:1611. 10.3390/jcm9061611 32466507 PMC7356286

[76] Manfrini O, Yoon J, van der Schaar M et al. Sex differences in modifiable risk factors and severity of coronary artery disease. J Am Heart Assoc 2020;9:e017235. 10.1161/JAHA.120.017235 32981423 PMC7792418

[77] Yusuf S, Hawken S, Ounpuu S et al. Effect of potentially modifiable risk factors associated with myocardial infarction in 52 countries (the INTERHEART study): case-control study. Lancet 2004;364:937–52. 10.1016/S0140-6736(04)17018-9 15364185

[78] Carrero JJ, de Mutsert R, Axelsson J et al. Sex differences in the impact of diabetes on mortality in chronic dialysis patients. Nephrol Dial Transplant 2011;26:270–6. 10.1093/ndt/gfq386 20621930

[79] Nakamura K, Nakagawa H, Murakami Y et al. Smoking increases the risk of all-cause and cardiovascular mortality in patients with chronic kidney disease. Kidney Int 2015;88:1144–52. 10.1038/ki.2015.212 26200944

[80] Shen Y, Cai R, Sun J et al. Diabetes mellitus as a risk factor for incident chronic kidney disease and end-stage renal disease in women compared with men: a systematic review and meta-analysis. Endocrine 2017;55:66–76. 10.1007/s12020-016-1014-6 27477292

[81] Koye DN, Shaw JE, Reid CM et al. Incidence of chronic kidney disease among people with diabetes: a systematic review of observational studies. Diabet Med 2017;34:887–901. 10.1111/dme.13324 28164387

[82] Piccoli GB, Alrukhaimi M, Liu ZH et al. Women and kidney diseases: questions unanswered and answers unquestioned. Kidney Int Rep 2018;3:225–35. 10.1016/j.ekir.2018.01.001 29725625 PMC5932302

[83] Barrett PM, McCarthy FP, Kublickiene K et al. Adverse pregnancy outcomes and long-term maternal kidney disease: a systematic review and meta-analysis. JAMA Netw Open 2020;3:e1920964. 10.1001/jamanetworkopen.2019.20964 32049292 PMC12527481

[84] Mosca L, Benjamin EJ, Berra K et al. Effectiveness-based guidelines for the prevention of cardiovascular disease in women—2011 update: a guideline from the American Heart Association. J Am Coll Cardiol 2011;57:1404–23. 10.1016/j.jacc.2011.02.005 21388771 PMC3124072

[85] Bailey AL, Scantlebury DC, Smyth SS. Thrombosis and antithrombotic therapy in women. Arterioscler Thromb Vasc Biol 2009;29:284–8. 10.1161/ATVBAHA.108.179788 19221205 PMC2801157

[86] DuPont JJ, Kenney RM, Patel AR et al. Sex differences in mechanisms of arterial stiffness. Br J Pharmacol 2019;176:4208–25. 10.1111/bph.14624 30767200 PMC6877796

[87] Coutinho T. Arterial stiffness and its clinical implications in women. Can J Cardiol 2014;30:756–64. 10.1016/j.cjca.2014.03.020 24970788

[88] Zoccali C, Vanholder R, Massy ZA et al. The systemic nature of CKD. Nat Rev Nephrol 2017;13:344–58. 10.1038/nrneph.2017.52 28435157

[89] Amdur RL, Feldman HI, Dominic EA et al. Use of measures of inflammation and kidney function for prediction of atherosclerotic vascular disease events and death in patients with CKD: findings from the CRIC study. Am J Kidney Dis 2019;73:344–53. 10.1053/j.ajkd.2018.09.012 30545708 PMC6812505

[90] Ward LJ, Laucyte-Cibulskiene A, Hernandez L et al. Coronary artery calcification and aortic valve calcification in patients with kidney failure: a sex-disaggregated study. Biol Sex Differ 2023;14:48. 10.1186/s13293-023-00530-x 37443048 PMC10347725

[91] Mauvais-Jarvis F, Bairey Merz N, Barnes PJ et al. Sex and gender: modifiers of health, disease, and medicine. Lancet 2020;396:565–82. 10.1016/S0140-6736(20)31561-0 32828189 PMC7440877

[92] Rodriguez-Montes L, Ovchinnikova S, Yuan X et al. Sex-biased gene expression across mammalian organ development and evolution. Science 2023;382:eadf1046. 10.1126/science.adf1046 37917687 PMC7615307

[93] Lopes-Ramos CM, Chen CY, Kuijjer ML et al. Sex differences in gene expression and regulatory networks across 29 Human tissues. Cell Rep 2020;31:107795. 10.1016/j.celrep.2020.107795 32579922 PMC7898458

[94] Mauvais-Jarvis F. Sex differences in energy metabolism: natural selection, mechanisms and consequences. Nat Rev Nephrol 2024:20:56–69. 10.1038/s41581-023-00781-2 37923858

[95] Sanz AB, Sanchez-Nino MD, Ramos AM et al. Regulated cell death pathways in kidney disease. Nat Rev Nephrol 2023;19:281–99. 10.1038/s41581-023-00694-0 36959481 PMC10035496

[96] Martin-Sanchez D, Ruiz-Andres O, Poveda J et al. Ferroptosis, but not necroptosis, is important in nephrotoxic folic acid-induced AKI. J Am Soc Nephrol 2017;28:218–29. 10.1681/ASN.2015121376 27352622 PMC5198282

[97] Ide S, Ide K, Abe K et al. Sex differences in resilience to ferroptosis underlie sexual dimorphism in kidney injury and repair. Cell Rep 2022;41:111610. 10.1016/j.celrep.2022.111610 36351395 PMC9795409

[98] Weger BD, Gobet C, Yeung J et al. The mouse microbiome is required for sex-specific diurnal rhythms of gene expression and metabolism. Cell Metab 2019;29:362–382 e8. 10.1016/j.cmet.2018.09.023 30344015 PMC6370974

[99] Langer A, Meleis A, Knaul FM et al. Women and Health: the key for sustainable development. Lancet 2015;386:1165–210. 10.1016/S0140-6736(15)60497-4 26051370

[100] Bruce R, Cavgias A, Meloni L et al. Under pressure: women's leadership during the COVID-19 crisis. J Dev Econ 2022;154:102761. 10.1016/j.jdeveco.2021.102761 34785851 PMC8581441

[101] Murphy A, Palafox B, Walli-Attaei M et al. The household economic burden of non-communicable diseases in 18 countries. BMJ Glob Health 2020;5:e002040. 10.1136/bmjgh-2019-002040 PMC7042605 32133191

[102] Pelletier R, Khan NA, Cox J et al. Sex versus gender-related characteristics: which predicts outcome after acute coronary syndrome in the young? J Am Coll Cardiol 2016;67:127–35. 10.1016/j.jacc.2015.10.067 26791057

[103] Ahmed SB, Dumanski SM. Do sex and gender matter in kidney and cardiovascular disease? Am J Kidney Dis 2021;78:177–9. 10.1053/j.ajkd.2021.05.002 34120781

[104] Goldberg R, Goff D, Cooper L et al. Age and sex differences in presentation of symptoms among patients with acute coronary disease: the REACT Trial. Rapid early action for coronary treatment. Coron Artery Dis 2000;11:399–407. 10.1097/00019501-200007000-00004 10895406

[105] Kaul P, Chang WC, Westerhout CM et al. Differences in admission rates and outcomes between men and women presenting to emergency departments with coronary syndromes. CMAJ 2007;177:1193–9. 10.1503/cmaj.060711 17984470 PMC2043078

[106] Manandhar M, Hawkes S, Buse K et al. Gender, health and the 2030 agenda for sustainable development. Bull World Health Organ 2018;96:644–53. 10.2471/BLT.18.211607 30262946 PMC6154065

[107] van Mil D, Kieneker LM, Evers-Roeten B et al. Participation rate and yield of two home-based screening methods to detect increased albuminuria in the general population in the Netherlands (THOMAS): a prospective, randomised, open-label implementation study. Lancet 2023;402:1052–64. 10.1016/S0140-6736(23)00876-0 37597522

[108] Hecking M, Hodlmoser S, Ahmed SB et al. The other way around: living with chronic kidney disease from the perspective of men. Semin Nephrol 2022;42:122–8. 10.1016/j.semnephrol.2022.04.003 35718360

[109] Seng JJB, Tan JY, Yeam CT et al. Factors affecting medication adherence among pre-dialysis chronic kidney disease patients: a systematic review and meta-analysis of literature. Int Urol Nephrol 2020;52:903–16. 10.1007/s11255-020-02452-8 32236780

[110] van de Luijtgaarden MWM, Caskey FJ, Wanner C et al. Uraemic symptom burden and clinical condition in women and men of ≥65 years of age with advanced chronic kidney disease: results from the EQUAL study. Nephrol Dial Transplant 2019;34:1189–96. 10.1093/ndt/gfy155 29905848

[111] Bai J, Yang JY, Di JK et al. Gender and socioeconomic disparities in global burden of chronic kidney disease due to glomerulonephritis: a global analysis. Nephrology (Carlton) 2023;28:159–67. 10.1111/nep.14137 36564906

[112] Valdivielso JM, Carriazo S, Martin M et al. Gender-specific risk factors and outcomes of hyperkalemia in CKD patients: smoking as a driver of hyperkalemia in men. Clin Kidney J 2023;17:sfad212. 10.1093/ckj/sfad212 PMC10768768 38186899

[113] Scholes-Robertson N, Viecelli AK, Tong A et al. Let's talk about sex ... and CKD. Clin J Am Soc Nephrol 2023;18:1092–4. 10.2215/CJN.0000000000000140 36888918 PMC10564341

[114] Tong A, Evangelidis N, Kurnikowski A et al. Nephrologists' Perspectives on gender disparities in CKD and dialysis. Kidney Int Rep 2022;7:424–35. 10.1016/j.ekir.2021.10.022 35257055 PMC8897691

[115] Luyckx VA, Harris DCH, Varghese C et al. Bringing equity in access to quality dialysis. Lancet 2021;398:10–11. 10.1016/S0140-6736(21)00732-7 33865498

[116] Pinho-Gomes A-C, Carcel C, Woodward M et al. Women's representation in clinical trials of patients with chronic kidney disease. Clin Kidney J 2023;16:1457–64. 10.1093/ckj/sfad018 37664564 PMC10469102

[117] Fernandez-Prado R, Ortiz A. A sudden decrease in serum creatinine and estimated glomerular filtration rate: clinical implications of administrative gender assignment in transgender persons. Clin Kidney J 2020;13:1107–8. 10.1093/ckj/sfz152 33391757 PMC7769535

